# Role of Vascular Adaptation in Determining Systolic Blood Pressure in Young Adults

**DOI:** 10.1161/JAHA.119.014375

**Published:** 2020-03-30

**Authors:** Shikai Yu, Jessica E. Middlemiss, Chiara Nardin, Stacey S. Hickson, Karen L. Miles, Yasmin, Kaisa M. Maki‐Petaja, Barry J. McDonnell, John R. Cockcroft, Ian B. Wilkinson, Carmel M. McEniery, the Enigma Study Investigators, Samantha Benedict, Zahid Dhakam, Lisa Day, Maggie Munnery, Pawan Pusalkar, Christopher Retallick, Ramsey Sabit, James Sharman, Jane Smith, Jean Woodcock‐Smith, Edna Thomas, Sharon Wallace

**Affiliations:** ^1^ Department of Cardiology Shanghai Tenth People’s Hospital Tongji University School of Medicine Shanghai China; ^2^ Division of Experimental Medicine and Immunotherapeutics University of Cambridge United Kingdom; ^3^ Dipartimento di Medicina (DIMED) University of Padova Italy; ^4^ Cardiff School of Health Sciences Cardiff Metropolitan University Cardiff United Kingdom

**Keywords:** cardiac output, hemodynamics, minimum forearm vascular resistance, peripheral vascular resistance, pulse pressure, High Blood Pressure, Hemodynamics, Mechanisms, Physiology

## Abstract

**Background:**

Two individuals can have a similar pulse pressure (PP) but different levels of systolic blood pressure (SBP), although the underlying mechanisms have not been described. We hypothesized that, for a given level of PP, differences in SBP relate to peripheral vascular resistance (PVR); and we tested this hypothesis in a large cohort of healthy young adults.

**Methods and Results:**

Demographic, biochemical, and hemodynamic data from 3103 subjects were available for the current analyses. In both men and women, for a given level of PP, higher SBP was associated with significantly higher body weight, body mass index, heart rate, and PVR (*P*<0.05 versus those with lower BP for all comparisons). Moreover, stratifying individuals by quartiles of PP and PVR revealed a stepwise increase in SBP from the lowest to highest quartile for each variable, with the highest SBP occurring in those in the highest quartile of both PP and PVR (*P*<0.001 for overall trend for both sexes). PVR was also increased with increasing tertile of minimum forearm vascular resistance, in both men (*P*=0.002) and women (*P*=0.03).

**Conclusions:**

Increased PVR, mediated in part through altered resistance vessel structure, strongly associates with the elevation of SBP for a given level of PP in young adults. An impaired ability to adapt PVR appropriately to a given level of PP may be an important mechanism underlying elevated SBP in young adults.

**Nonstandard Abbreviations and Acronyms**
AIx: augmentation index
aPWV: aortic pulse wave velocity
BMI: body mass index
MAP: mean arterial pressure
MFVR: minimum forearm vascular resistance
PP: pulse pressure
PPA: pulse pressure amplification
PVR: peripheral vascular resistance
SBP: systolic blood pressure
TAC: total arterial compliance

**Clinical Perspective**

**What Is New?**

- Elevated peripheral vascular resistance is the major mechanism explaining why differences in systolic blood pressure occur between young adults presenting with similar levels of pulse pressure and stroke volume.
- The differences in peripheral vascular resistance are mediated, in part, through altered resistance vessel structure and are also associated with greater body weight and body mass index.

**What Are the Clinical Implications?**

- High systolic blood pressure in young adults suggests that the vascular adaptation to a given level of stroke volume is impaired, and this impairment is associated with alterations in the underlying vascular structure.

The most common form of hypertension in young adults is isolated systolic hypertension (ISH).^1^, ^2^, ^3^ Although often considered an innocuous condition,^4^ recent evidence demonstrates that ISH in young to middle‐aged adults is associated with an increased risk of cardiovascular mortality in later life.^5^ Moreover, data from the FHS (Framingham Heart Study) show that elevated pulse pressure (PP), which characterizes ISH, tracks from young adulthood throughout life, and is associated with increased mortality in older adults.^6^

The key hemodynamic determinants of PP are stroke volume and aortic stiffness, although in young adults, it is stroke volume rather than aortic stiffness that is predominantly associated with increased PP, particularly in men.^3^ However, 2 individuals can have similarly wide PP, but different levels of systolic blood pressure (SBP), suggesting differences in the adaptive or maladaptive responses to high stroke volume. This is important clinically, because it may result in one individual being classified as normotensive and another hypertensive, with the potential for life‐long differences between them in terms of follow‐up BP monitoring and assessment of long‐term risk.

Systolic and diastolic BP values oscillate around mean arterial pressure (MAP), which is determined, physiologically, by cardiac output and peripheral vascular resistance (PVR). Therefore, we hypothesized that for a given level of PP, variations in SBP between individuals are related to PVR, and the aim of this study was to test this hypothesis in a large cohort of healthy young adults. In addition, we measured minimum forearm vascular resistance (MFVR) in a random subset of subjects to determine whether structural differences in the resistance vasculature were associated with variations in PVR.

## Methods

The data that support the findings of this study are available from the corresponding author on reasonable request.

### Study Population

Subjects were drawn from The Enigma Study population, which consists of 3145 individuals aged between 18 and 40 years, selected at random from 2 university populations in the United Kingdom (Cambridge and Wales; response rate ≈70%).^3^, ^7^ Subjects with diabetes mellitus, secondary forms of hypertension, and evidence of cardiovascular, renal, or systemic inflammatory diseases were excluded, as were subjects with serum cholesterol of ≥6.5 mmol/L or receiving any vasoactive medication. In the present analysis, 42 subjects with incomplete hemodynamic data were excluded; therefore, 3103 subjects were included in the final analysis. Approval for the study was obtained from the Local Research Ethics Committees, and written informed consent was obtained from each participant.

### Protocol

Each participant completed a detailed lifestyle and medical history questionnaire. Height and weight were measured; body surface area and body mass index (BMI) were calculated. After 20 minutes of supine rest, brachial BP and radial artery waveforms were recorded, as well as aortic pulse wave velocity (aPWV). Stroke volume and cardiac output were assessed, and PVR was calculated. Blood samples were collected from the antecubital vein under fasting conditions. Total cholesterol, low‐density lipoprotein cholesterol, high‐density lipoprotein cholesterol, triglycerides, and serum glucose were assessed using standard laboratory methods in an accredited laboratory. MFVR was assessed in a random subset of individuals.

### Hemodynamics

Brachial BP was measured in the dominant arm, using a validated oscillometric sphygmomanometer and an appropriately sized cuff (HEM‐705CP; Omron Corporation, Japan), while participants rested in a supine position. Three readings were obtained, and the mean value was used for analysis. A high‐fidelity micromanometer (SPC‐301; Millar Instruments), interfaced with a computer using SphygmoCor software (SphygmoCor, AtCor Medical, Australia), was applied to evaluate central BP, augmentation index (AIx), and aPWV. In brief, radial artery waveforms were recorded, and pulse wave analysis was used to generate a corresponding central (ascending aortic) waveform. From this, central BP, MAP, AIx, and heart rate were derived. PP amplification (PPA) was calculated as the ratio between brachial and central PP. Pulse wave recordings were then performed consecutively at the common carotid and femoral arteries to assess aPWV. Path length was calculated as the superficial distance from the sternal notch to the femoral pulse site minus the sternal notch to the carotid pulse site distance, using a tape measure.

Stroke volume and cardiac output were measured using a validated noninvasive, inert gas rebreathing technique, which has been validated against thermodilution and direct Fick methods for measurement of pulmonary blood flow and cardiac output^8^, ^9^, ^10^ and is reproducible and sensitive to the influence of body size.^11^ In short, while resting, subjects continuously rebreathed a gas mixture (1% SF_6_, 5% N_2_O, and 94% O_2_) over 20 seconds, with a breathing rate of 15/min. Expired gases were sampled continuously and analyzed by an infrared photoacoustic gas analyzer, for the determination of stroke volume and cardiac output. PVR was calculated using the formula: PVR (dynes·s·cm^−5^)=MAP (mm Hg)×80/cardiac output (mL/min), and total arterial compliance (TAC) was defined as the ratio of stroke volume/central PP.^12^ All measurements were performed by trained study personnel.

### Assessment of MFVR

MFVR was assessed in 263 randomly scheduled individuals. Venous occlusion plethysmography (EC6; Hokanson Inc, WA) was undertaken in the nondominant arm to measure forearm blood flow before and after 13 minutes of ischemia, as previously described in detail.^13^, ^14^ Forearm vascular resistance was calculated as the MAP divided by forearm blood flow. The minimum vascular resistance provides an indirect measure of resistance vessel structure and allowed us to examine whether differences in resistance vessel structure were associated with variations in PVR.

### Statistical Analysis

Continuous variables are presented as means±SD, and categorical variables are presented as absolute numbers (percentages). All analyses were performed separately for men and women because of the significant differences in hemodynamics and mechanisms of BP elevation between sexes in young adults. Three strategies were adopted to examine our hypothesis that for a given level of PP, variations in SBP between individuals are related to PVR. Participants were initially grouped according to quartile of brachial PP, with each PP quartile further stratified by tertile of brachial SBP. This approach ensured adequate separation of hemodynamic data across PP quartiles, while retaining relatively large sample sizes in each SBP group. For the primary analyses, we chose to focus on 4 groups within each sex, comprising the extremes of PP (lowest and highest PP quartiles) and their corresponding lowest and highest SBP tertiles, to allow us to examine, in detail, the hemodynamic mechanisms underlying SBP elevation within a given level of PP. One‐way ANCOVA was used to explore differences in demographic, biochemical, and hemodynamic variables among the 4 groups, with age included as a covariate in all models (except the age model). Height and heart rate were additionally included as covariates in the AIx/PPA models, MAP and heart rate were included in the aPWV model, and BMI and heart rate were included in the TAC model. Post hoc analyses were conducted using the Bonferroni method. Additional analyses, based on all 4 PP quartiles and all corresponding SBP tertiles (12 comparator groups in total), were also undertaken. Second, using the entire cohort, men and women were grouped by quartile of PVR and PP and an ANOVA model was constructed with SBP as the dependent variable and PVR/PP quartile as the independent variable for analysis of the overall trend. Third, treating all data as continuous variables, univariable and multivariable linear regression models were constructed to investigate the association of SBP with each hemodynamic determinant of BP. Models were constructed for the whole cohort, and the lowest and highest PP groups. Models for the middle 2 quartiles of PP are shown as a supplementary analysis. Finally, in a separate analysis, men and women were grouped according to tertile of MFVR. One‐way ANOVA was used to explore differences in PVR between MFVR tertiles for each sex, with PVR as the dependent variable and MFVR group (3 groups representing the lowest to highest tertiles of MFVR) as the independent variable, for analysis of the overall trend. Statistical analysis was performed using SAS software, version 9.4 (SAS Institute, Cary, NC). Statistical significance was defined as *P*<0.05.

## Results

Characteristics of the study participants are presented in Table 1. The ranges of PP for each quartile, and SBP for each tertile, are shown in Tables S1 and S2. Men and women were similar in terms of age and low‐density lipoprotein cholesterol, but differed significantly in other demographic, biochemical, and hemodynamic parameters. In particular, men had significantly higher levels of BP, cardiac output, stroke volume, TAC, PPA, and aPWV, but lower heart rate, AIx, and PVR than women.

**Table 1:** Characteristics of Participants

Variables	Total Population			
	Total (n=3103)	Men (n=1539)	Women (n=1564)	*P* Value
Age, y	23±6	23±6	23±6	0.94
Height, m	1.72±0.10	1.79±0.07	1.65±0.07	<0.001
Weight, kg	70.7±15.0	77.9±14.2	63.5±12.1	<0.001
Body mass index, kg/m^2^	23.8±4.1	24.4±4.1	23.2±4.1	<0.001
Body surface area, m^2^	1.83±0.21	1.96±0.18	1.70±0.16	<0.001
Smoker, n (%)	316 (10.2)	180 (11.7)	136 (8.7)	0.004
Total cholesterol, mmol/L	4.18±0.92	4.10±0.96	4.26±0.86	<0.001
LDL cholesterol, mmol/L	2.33±0.79	2.30±0.82	2.36±0.76	0.07
HDL cholesterol, mmol/L	1.44±0.38	1.33±0.33	1.54±0.39	<0.001
Triglycerides, mmol/L	1.03±0.75	1.17±0.90	0.90±0.53	<0.001
Glucose, mmol/L	4.68±1.98	4.83±2.72	4.55±0.75	0.002
Brachial SBP, mm Hg	118±15	126±12	111±13	<0.001
Brachial DBP, mm Hg	70±10	71±9	69±10	<0.001
Brachial PP, mm Hg	49±11	55±9	42±8	<0.001
Central SBP, mm Hg	101±13	105±11	97±13	<0.001
Central PP, mm Hg	30±7	34±6	27±6	<0.001
MAP, mm Hg	84±11	86±10	83±11	<0.001
Heart rate, beats/min	67±11	66±11	68±11	<0.001
PPA	1.61±0.16	1.63±0.14	1.59±0.18	<0.001
AIx, %	3.74±12.97	0.43±11.64	7.02±13.39	<0.001
aPWV, m/s	5.76±0.98	5.93±0.98	5.60±0.95	<0.001
Cardiac output, L/min	7.31±2.05	8.25±2.09	6.39±1.52	<0.001
Cardiac index, L/min per m^2^	3.99±0.94	4.21±0.99	3.76±0.83	<0.001
Stroke volume, mL/beat	98±29	111±30	85±21	<0.001
Stroke volume index, mL/beat per m^2^	53±13	57±14	50±12	<0.001
PVR, dyne·s·cm^−5^	995±310	897±285	1092±304	<0.001
TAC, mL/beat per mm Hg	3.29±0.93	3.33±0.94	3.24±0.92	0.01

Values are means±SD or number (percentage). Values in the final column represent results of comparison between men and women. AIx indicates augmentation index; aPWV, aortic pulse wave velocity; DBP, diastolic blood pressure; HDL, high‐density lipoprotein; LDL, low‐density lipoprotein; MAP, mean arterial pressure; PP, pulse pressure; PPA, PP amplification; PVR, peripheral vascular resistance; SBP, systolic blood pressure; and TAC, total arterial compliance.

### Demographic and Biochemical Differences Between SBP Groups

Comparisons of demographic, biochemical, and hemodynamic characteristics between lower and higher SBP groups within the lowest and highest quartiles of PP are shown in Tables 2 and 3, for men and women, respectively. For a given level of PP (in either the lowest or highest quartile) and after adjustment for age, men with higher SBP were heavier and had higher BMI, total cholesterol, low‐density lipoprotein cholesterol, triglycerides, and glucose than those with lower SBP. Women with higher SBP were also heavier and had a higher BMI than those with lower SBP, but there were no significant differences in biochemical parameters.

**Table 2:** Comparison of Variables Between Individuals With Higher Versus Lower SBP in Men

Variables	Lowest PP Quartile (n=383)		Highest PP Quartile (n=395)		ANCOVA *P* Value
	Lower SBP (n=130)	Higher SBP (n=127)	Lower SBP (n=126)	Higher SBP (n=130)	
Demographic and biochemical variables					
Age, y	22±5	27±7^a^	21±3	24±6^b^	<0.001
Height, m	1.76±0.08	1.77±0.07	1.81±0.06^a^	1.80±0.07^c^	<0.001
Weight, kg	69.0±12.8	82.0±16.7^a^	77.2±9.8^a^	85.9±15.6^b^	<0.001
Body mass index, kg/m^2^	22.4±3.8	26.1±4.8^a^	23.6±2.5	26.5±4.5^b^	<0.001
Body surface area, m^2^	1.84±0.18	1.99±0.20^a^	1.97±0.14^a^	2.05±0.18^b^, ^c^	<0.001
Total cholesterol, mmol/L	3.99±0.84	4.59±1.13^a^	3.83±0.89	4.33±1.13^b^	<0.001
LDL cholesterol, mmol/L	2.29±0.77	2.71±1.00^a^	2.10±0.76	2.42±1.05^b^, ^c^	<0.001
HDL cholesterol, mmol/L	1.33±0.31	1.29±0.35	1.30±0.30	1.36±0.40	0.40
Triglycerides, mmol/L	0.93±0.51	1.37±0.92^a^	1.18±0.85	1.39±1.01	<0.001
Glucose, mmol/L	4.52±0.80	4.81±0.87^a^	4.82±0.84^a^	4.98±0.76	<0.001
Hemodynamic variables					
Brachial SBP, mm Hg	106±5	127±8^a^	125±4^a^	149±10^b^, ^c^	<0.001
Brachial DBP, mm Hg	63±5	82±8^a^	62±4	79±9^b^, ^c^	<0.001
Brachial PP, mm Hg	43±4	45±3^a^	64±3^a^	70±8^b^, ^c^	<0.001
Central SBP, mm Hg	91±5	112±9**	101±4^a^	123±11^b^, ^c^	<0.001
Central PP, mm Hg	27±3	29±3^a^	39±3^a^	42±6^b^, ^c^	<0.001
MAP, mm Hg	76±5	96±9^a^	79±5^a^	99±10^b^, ^c^	<0.001
Heart rate, beats/min	61±10	70±10^a^	63±10	70±13^b^	<0.001
PPA	1.62±0.14	1.58±0.18	1.66±0.11	1.66±0.15^c^	<0.001
Adjusted PPA^d^	1.63±0.10	1.59±0.11^a^	1.65±0.11	1.64±0.11^c^	<0.001
AIx, %	1.12±10.61	6.84±12.95^a^	−3.91±10.57^a^	−0.01±12.56^b^, ^c^	<0.001
Adjusted AIx, %^d^	0.30±11.29	5.20±11.38^a^	−2.03±11.22	1.19±10.95^c^	<0.001
aPWV, m/s	5.47±0.77	6.44±1.18^a^	5.49±0.71	6.63±1.23^b^	<0.001
Adjusted aPWV, m/s^e^	6.17±1.14	5.82±1.01	6.10±1.01	5.92±1.04	0.16
Cardiac output, L/min	6.98±1.65	7.68±2.01^a^	9.21±2.02^a^	9.56±2.05^c^	<0.001
Cardiac index, L/min per m^2^	3.80±0.83	3.87±0.98	4.67±0.96^a^	4.67±0.98^c^	<0.001
Stroke volume, mL/beat	103±25	99±28	124±32^a^	121±30^c^	<0.001
Stroke volume index, mL/beat per m^2^	56±13	50±13^a^	63±15^a^	59±15^c^	<0.001
PVR, dyne·s·cm^−5^	921±237	1075±305^a^	722±202^a^	874±231^b^, ^c^	<0.001
TAC, mL/beat per mm Hg	3.93±1.00	3.48±1.04^a^	3.23±0.82^a^	2.85±0.71^b^, ^c^	<0.001
Adjusted TAC, mL/beat per mm Hg^f^	3.92±1.02	3.58±1.01^a^	3.14±0.90^a^	2.84±0.91^c^	<0.001

Values are means±SD. AIx indicates augmentation index; aPWV, aortic pulse wave velocity; DBP, diastolic blood pressure; HDL, high‐density lipoprotein; LDL, low‐density lipoprotein; MAP, mean arterial pressure; PP, pulse pressure; PPA, PP amplification; PVR, peripheral vascular resistance; SBP, systolic blood pressure; and TAC, total arterial compliance.

a *P*<0.05 vs lowest PP quartile and lower SBP.

b *P*<0.05 vs highest PP quartile and lower SBP.

c *P*<0.05 vs lowest PP quartile and higher SBP.

d Further adjusted for height and heart rate.

e Further adjusted for MAP and heart rate.

f Further adjusted for body mass index and heart rate.

**Table 3:** Comparison of Variables Between Individuals With Higher Versus Lower SBP in Women

Variables	Lowest PP Quartile (n=427)		Highest PP Quartile (n=406)		ANCOVA *P* Value
	Lower SBP (n=137)	Higher SBP (n=145)	Lower SBP (n=134)	Higher SBP (n=134)	
Demographic and biochemical variables					
Age, y	22±4	24±6	22±5	26±7^a^, ^b^	<0.001
Height, m	1.63±0.07	1.63±0.07	1.67±0.06^c^	1.65±0.08^a^	<0.001
Weight, kg	57.4±11.2	63.7±13.3^c^	63.8±7.9^c^	71.5±17.0^a^, ^b^	<0.001
Body mass index, kg/m^2^	21.5±3.8	23.9±4.5^c^	22.8±2.7	26.3±5.9^a^, ^b^	<0.001
Body surface area, m^2^	1.61±0.16	1.68±0.17^c^	1.72±0.11^c^	1.78±0.20^a^, ^b^	<0.001
Total cholesterol, mmol/L	4.13±0.84	4.35±0.79	4.21±0.80	4.43±0.86	0.15
LDL cholesterol, mmol/L	2.30±0.64	2.46±0.71	2.28±0.69	2.49±0.79	0.26
HDL cholesterol, mmol/L	1.54±0.40	1.50±0.40	1.54±0.36	1.50±0.42	0.40
Triglycerides, mmol/L	0.77±0.40	0.94±0.52	0.93±0.60	1.10±0.72	0.001
Glucose, mmol/L	4.36±0.75	4.56±0.78	4.50±0.71	4.72±0.83	0.004
Hemodynamic variables					
Brachial SBP, mm Hg	93±5	113±7^c^	110±4^c^	138±12^a^, ^b^	<0.001
Brachial DBP, mm Hg	62±5	78±8^c^	60±4	83±11^a^, ^b^	<0.001
Brachial PP, mm Hg	32±3	34±3^c^	50±3^c^	55±8^a^, ^b^	<0.001
Central SBP, mm Hg	82±5	102±8^c^	92±4^c^	121±15^a^, ^b^	<0.001
Central PP, mm Hg	20±3	22±3^c^	31±3^c^	37±8^a^, ^b^	<0.001
MAP, mm Hg	72±5	90±80^c^	75±4^c^	102±13^a^, ^b^	<0.001
Heart rate, beats/min	64±11	70±11^c^	64±10	73±11^a^	<0.001
PPA	1.57±0.17	1.55±0.19	1.63±0.13^c^	1.50±0.23^a^, ^b^	<0.001
Adjusted PPA^d^	1.58±0.16	1.54±0.15	1.62±0.16	1.51±0.16^a^	<0.001
AIx, %	6.76±12.94	12.16±12.29^c^	1.17±11.95	15.18±15.16^a^	<0.001
Adjusted AIx, %^d^	6.00±11.70	12.39±11.32^c^	2.55±11.69	14.39±12.04^a^	<0.001
aPWV, m/s	5.27±0.94	5.80±0.82^c^	5.25±0.70	6.48±1.23^a^, ^b^	<0.001
Adjusted aPWV, m/s^e^	5.84±1.05	5.58±0.84	5.70±0.93	5.63±1.15	0.20
Cardiac output, L/min	5.63±1.29	6.06±1.36^c^	6.66±1.56^c^	6.96±1.71^b^	<0.001
Cardiac index, L/min per m^2^	3.48±0.69	3.61±0.77	3.88±0.88^c^	3.93±0.94^b^	<0.001
Stroke volume, mL/beat	80±19	77±17	92±24^c^	86±25^b^	<0.001
Stroke volume index, mL/beat per m^2^	50±11	46±9^c^	54±14^c^	49±14^a^	<0.001
PVR, dyne·s·cm^−5^	1070±255	1244±298^c^	952±248^c^	1252±399^a^	<0.001
TAC, mL/beat per mm Hg	3.97±0.95	3.49±0.99^c^	3.01±0.79^c^	2.39±0.49^a^, ^b^	<0.001
Adjusted TAC, mL/beat per mm Hg^f^	3.98±0.94	3.50±0.96^c^	2.97±0.93^c^	2.40±0.98^a^, ^b^	<0.001

Values are means±SD. AIx indicates augmentation index; aPWV, aortic pulse wave velocity; DBP, diastolic blood pressure; HDL, high‐density lipoprotein; LDL, low‐density lipoprotein; MAP, mean arterial pressure; PP, pulse pressure; PPA, PP amplification; PVR, peripheral vascular resistance; SBP, systolic blood pressure; and TAC, total arterial compliance.

a *P*<0.05 vs highest PP quartile and lower SBP.

b *P*<0.05 vs lowest PP quartile and higher SBP.

c *P*<0.05 vs lowest PP quartile and lower SBP.

d Further adjusted for height and heart rate.

e Further adjusted for MAP and heart rate.

f Further adjusted for body mass index and heart rate.

### Hemodynamic Differences Between SBP Groups

In both men (Table 2) and women (Table 3), for a given level of PP, those with higher SBP had a higher MAP and heart rate versus those with lower SBP. Cardiac output was not consistently higher in those individuals with higher SBP, and adjusting for body surface area attenuated any differences between SBP groups. In contrast, PVR, AIx, and aPWV were all markedly elevated, and TAC was significantly lower, in subjects with higher SBP, although the significant difference in aPWV between SBP groups was eliminated after further adjustment for MAP and heart rate. In addition, central SBP, but not PPA, was higher in those with higher brachial SBP irrespective of the level of PP. Consistent results were obtained when all PP and SBP groups were considered, rather than just the extremes (Tables S3 and S4).

### Influence of PP and PVR on SBP

**Figure 1:**
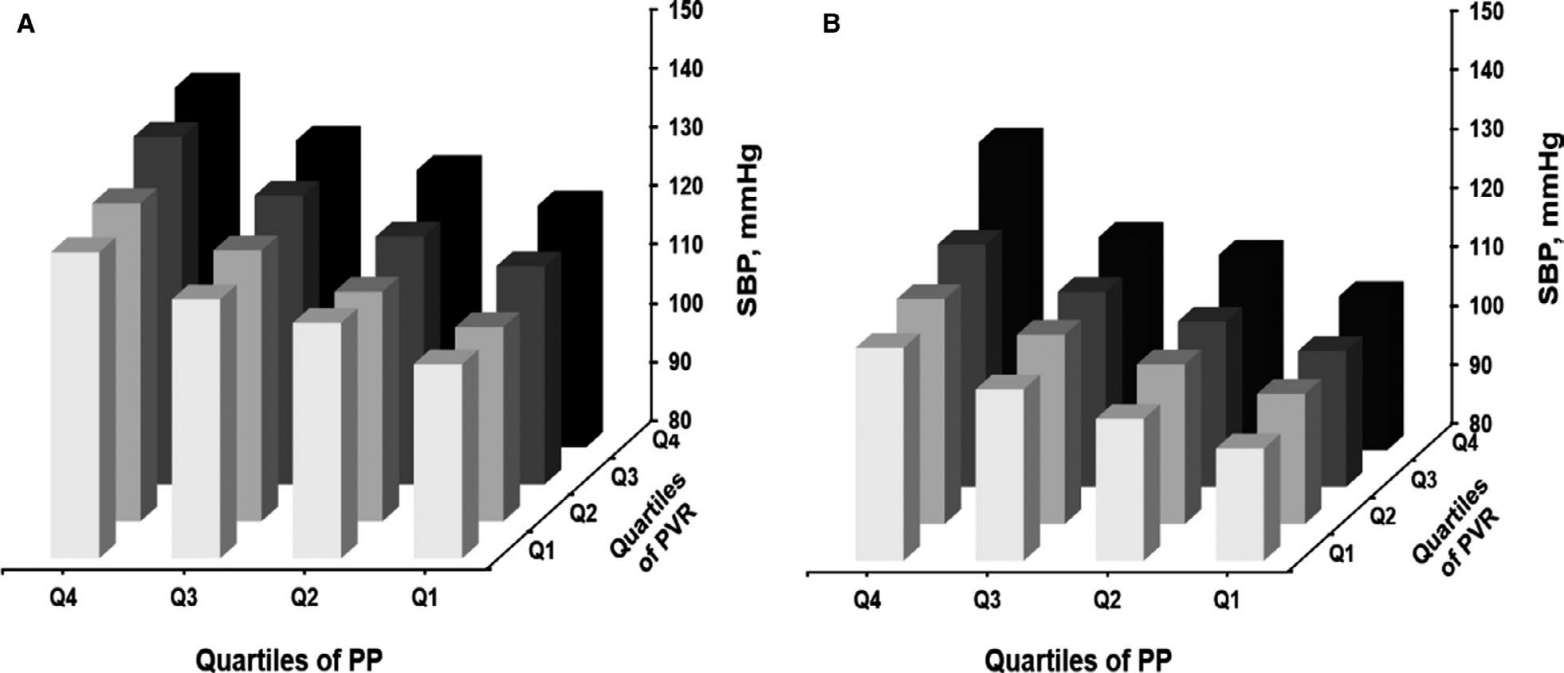
The influence of peripheral vascular resistance (PVR) on systolic blood pressure (SBP) at given levels of pulse pressure (PP) in men (**A**) and women (**B**). Q indicates quartile.

Using the entire cohort, men and women were stratified, separately, by quartile of PP and PVR, to investigate their influence on SBP (Figure 1). There was a graded increase in SBP from the lowest to the highest quartile of PP and PVR, for both men and women (*P*<0.001 for trend in both sexes), indicating that at any given level of PP, SBP increased with an increase in PVR. The highest level of SBP occurred in those individuals in the top quartile of PVR and PP. In multivariable linear regression analyses, independent associations of PVR, TAC, and AIx with SBP were consistently observed in various subgroups stratified by sex and PP (Tables 4 and 5 and Tables S5 and S6, for men and women, respectively).

**Table 4:** Association of SBP With Hemodynamic Parameters in Men

Models	Whole Population (n=1539)			Lowest PP Quartile (n=383)			Highest PP Quartile (n=395)		
	β	*P* Value	Model *R* ^2^	β	*P* Value	Model *R* ^2^	β	*P* Value	Model *R* ^2^
Univariable linear regression analysis									
Cardiac output	0.26	<0.001	0.07	0.10	0.05	0.01	0.14	0.004	0.02
Stroke volume	0.10	<0.001	0.01	−0.07	0.16	0.01	−0.04	0.45	0.002
PVR	0.09	<0.001	0.01	0.32	<0.001	0.10	0.27	<0.001	0.07
TAC	−0.30	<0.001	0.09	−0.22	<0.001	0.05	−0.22	<0.001	0.05
PPA	0.03	0.34	0.001	−0.16	0.003	0.03	−0.02	0.65	0.001
AIx	0.07	0.007	0.01	0.28	<0.001	0.08	0.19	<0.001	0.04
aPWV	0.40	<0.001	0.16	0.48	<0.001	0.24	0.53	<0.001	0.29
Multivariable linear regression analysis									
Cardiac output^a^	0.25	<0.001	0.18	0.08	0.08	0.20	0.14	0.002	0.21
Stroke volume^b^	0.15	<0.001	0.20	0.001	0.98	0.30	0.04	0.41	0.28
PVR^b^	0.07	0.006	0.18	0.26	<0.001	0.36	0.20	<0.001	0.31
TAC^b^	−0.28	<0.001	0.26	−0.15	0.001	0.32	−0.15	0.001	0.31
PPA^c^	−0.01	0.76	0.12	−0.16	0.003	0.28	−0.15	0.007	0.26
AIx^c^	0.08	0.005	0.12	0.22	<0.001	0.30	0.19	<0.001	0.26
aPWV^d^	0.004	0.84	0.66	−0.02	0.43	0.91	0.003	0.91	0.81

Univariable and multivariable linear regression analyses were performed. Variables that were not significantly associated with SBP in univariable models were excluded from multivariable analyses. β indicates standardized regression coefficient; AIx, augmentation index; aPWV, aortic pulse wave velocity; PP, pulse pressure; PPA, PP amplification; PVR, peripheral vascular resistance; SBP, systolic blood pressure; and TAC, total arterial compliance.

a Adjusted for age and body mass index.

b Adjusted for age, body mass index, and heart rate.

c Adjusted for age, height, and heart rate.

d Adjusted for age, body mass index, mean blood pressure, and heart rate.

**Table 5:** Association of SBP With Hemodynamic Parameters in Women

Models	Whole Population (n=1564)			Lowest PP Quartile (n=427)			Highest PP Quartile (n=406)		
	β	*P* Value	Model *R* ^2^	β	*P* Value	Model *R* ^2^	β	*P* Value	Model *R* ^2^
Univariable linear regression analysis									
Cardiac output	0.23	<0.001	0.05	0.19	<0.001	0.04	0.04	0.45	0.001
Stroke volume	0.07	0.004	0.01	−0.05	0.30	0.003	−0.06	0.23	0.004
PVR	0.23	<0.001	0.05	0.26	<0.001	0.07	0.47	<0.001	0.22
TAC	−0.40	<0.001	0.16	−0.24	<0.001	0.06	−0.36	<0.001	0.13
PPA	−0.21	<0.001	0.04	−0.10	0.04	0.01	−0.44	<0.001	0.19
AIx	0.25	<0.001	0.06	0.20	<0.001	0.04	0.52	<0.001	0.27
aPWV	0.47	<0.001	0.22	0.29	<0.001	0.09	0.66	<0.001	0.44
Multivariable linear regression analysis									
Cardiac output^a^	0.18	<0.001	0.18	0.10	0.03	0.12	0.05	0.25	0.26
Stroke volume^b^	0.10	<0.001	0.20	−0.05	0.30	0.19	−0.01	0.83	0.30
PVR^b^	0.22	<0.001	0.24	0.32	<0.001	0.28	0.37	<0.001	0.42
TAC^b^	−0.36	<0.001	0.31	−0.25	<0.001	0.24	−0.24	<0.001	0.35
PPA^c^	−0.26	<0.001	0.16	−0.17	0.002	0.15	−0.43	<0.001	0.39
AIx^c^	0.27	<0.001	0.18	0.26	<0.001	0.19	0.46	<0.001	0.41
aPWV^d^	0.03	0.06	0.77	0.01	0.78	0.86	0.09	<0.001	0.89

Univariable and multivariable linear regression analyses were performed. Variables that were not significantly associated with SBP in univariable models were excluded from multivariable analyses. β indicates standardized regression coefficient; AIx, augmentation index; aPWV, aortic pulse wave velocity; PP, pulse pressure; PPA, PP amplification; PVR, peripheral vascular resistance; SBP, systolic blood pressure; and TAC, total arterial compliance.

a Adjusted for age and body mass index.

b Adjusted for age, body mass index, and heart rate.

c Adjusted for age, height, and heart rate.

d Adjusted for age, body mass index, mean blood pressure, and heart rate.

### Influence of MFVR on PVR

**Figure 2:**
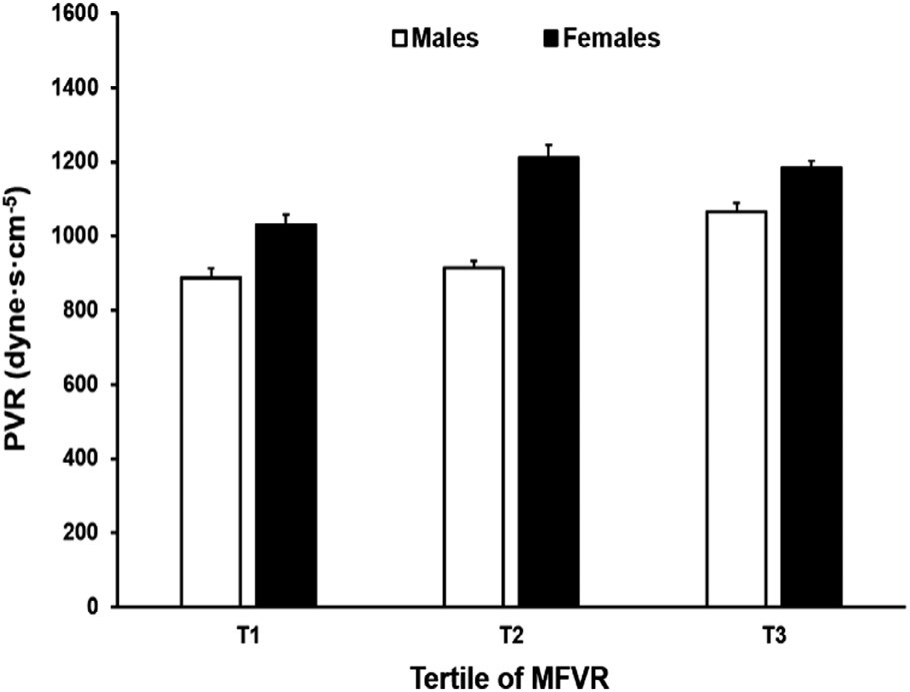
The influence of minimum forearm vascular resistance (MFVR) on peripheral vascular resistance (PVR) in men and women. Bars represent SEs. *P*=0.002 (men) and *P*=0.03 (women) for trend. T indicates tertile.

The detailed characteristics of 263 individuals in whom MFVR was measured are shown in Table 6. As MFVR increased from the lowest to the highest tertile, so too did PVR, in both men (*P* for trend=0.002) and women (*P* for trend=0.03) (Figure 2).

**Table 6:** Characteristics of the Subset of 263 Individuals With MFVR

Variables	Total (n=263)	Men (n=147)	Women (n=116)	*P* Value
Age, y	25.8±5.9	26.1±6.0	25.4±5.8	0.30
Body mass index, kg/m^2^	24.0±4.0	24.5±3.3	23.4±4.6	0.05
Systolic blood pressure, mm Hg	120±14	127±12	112±13	<0.001
Diastolic blood pressure, mm Hg	70±10	70±10	69±8	0.26
Pulse pressure, mm Hg	50±10	56±9	43±8	<0.001
Mean arterial pressure, mm Hg	85±11	87±11	82±10	<0.001
Heart rate, beats/min	64±11	62±10	67±11	<0.001
Augmentation index, %	8.29±11.9	6.33±12.46	10.77±10.75	0.003
aPWV, m/s	5.81±0.96	5.91±0.89	5.69±1.03	0.07
Cardiac output, L/min	7.01±1.89	7.77±1.92	6.06±1.34	<0.001
Stroke volume, mL	100±28	113±27	84±18	<0.001
PVR, dyne·s·cm^−5^	1038±304	955±281	1143±302	<0.001
MFVR, mm Hg/mL per min/100 mL	2.94±1.32	2.62±0.93	3.35±1.61	<0.001

Values are means±SD. Values in the final column represent results of comparison between men and women. aPWV indicates aortic pulse wave velocity; MFVR, minimum forearm vascular resistance; and PVR, peripheral vascular resistance.

## Discussion

We have demonstrated that elevated PVR was associated with elevated SBP for a given level of PP, in both men and women. Moreover, elevated PVR was associated with structural differences in resistance vessels in both men and women. Taken together, these data suggest that increased PVR, mediated in part through altered resistance vessel structure, represents an early maladaptive mechanism underlying the development of high SBP in young adults.

Elevated PP commonly occurs in elderly individuals, manifesting as ISH and typically caused by increased arterial stiffness.^15^, ^16^ However, elevated PP and ISH are also often present in young adults and particularly men, but are typically driven by an elevated stroke volume.^3^, ^17^, ^18^, ^19^ However, some individuals may have an elevated stroke volume and PP, but operate at a much lower SBP, and thus remain normotensive. In the current study, we have compared young adults with similar levels of PP, but significantly different levels of SBP, separately for men and women. We observed that PVR was significantly higher in those individuals with higher SBP, irrespective of whether PP was high or low. This was confirmed in analyses using the entire cohort where, at any given level of PP, higher PVR was associated with higher SBP and in multivariable regression analyses where PVR remained independently associated with SBP. The presence of a higher SBP could, therefore, represent an impaired ability of the resistance vasculature to respond appropriately to a given level of PP in young adults. More important, although some of the individuals with higher SBP may not yet be regarded as having established hypertension, they may be more likely to develop sustained hypertension in the future because both SBP and PP track strongly throughout life.^6^, ^20^, ^21^

Interestingly, in the individuals with higher SBP, increased PVR was accompanied by a higher body weight and greater BMI, irrespective of the level of PP. This suggests that increased body size may mediate an attenuated ability of the resistance vasculature to adapt appropriately to the higher levels of stroke volume and cardiac output also observed in these individuals. However, PVR was actually highest in those individuals with the lowest PP, and greater in women than men, irrespective of the level of SBP. In the current study, stroke volume and cardiac output were also lower in those individuals with lower PP and in women versus men, suggesting that PVR is the dominant hemodynamic determinant of BP in these individuals. The observations also support the notion that an insufficient reduction in PVR, in the face of an elevated stroke volume and cardiac output, will be associated with elevated SBP.

Differences in PVR may be mediated by structural or functional mechanisms in the resistance vessels. We have demonstrated that MFVR, which provides a measure of the structural characteristics of forearm resistance vessels, is positively associated with PVR. These data suggest that the elevated PVR observed in individuals with higher SBP is mediated, at least in part, through structural differences in the resistance vasculature. Interestingly, vascular remodeling and increased wall/lumen ratio are generally associated with middle‐aged or older individuals with established essential hypertension.^22^, ^23^ However, the average age of participants in the current study was only 23 years. Therefore, it is possible that inherent differences, or early changes, in resistance vessel structure may represent one of the initial hemodynamic derangements that predispose to the development of elevated BP in young adults, especially when combined with an elevated stroke volume arising from a large body size or some other cardiogenic mechanism. It may, of course, be the case that structural differences in the resistance vasculature could have been present from birth or early life. The differences in PVR might also be related, in part, to functional differences in the regulation of autonomic tone or vasoactive mediators, although further investigations are required to explore these possibilities.

In addition to PVR, we also observed differences in heart rate, AIx, and TAC between higher and lower SBP groups, suggesting that these parameters might also define SBP for a given level of PP. On the basis of animal experiments, Stergiopulos and Westerhof^24^ demonstrated that, for a given cardiac ejection pattern (aortic flow), PP is determined by PVR and TAC. In the present study, we have confirmed these findings in humans and identified an influence of wave reflections. Intriguingly, we also observed an independent association between aPWV and SBP in women in the highest quartile of PP, suggesting that aortic stiffening contributes to elevated SBP in young women.

Substantial PPA from heart to peripheral arteries is frequently observed in young adults, which may underlie the variation of SBP for a given level of PP. However, in our study, it was central SBP, rather than PPA, that was elevated in those individuals with high SBP. Moreover, at each level of PP, PPA was inversely associated with elevated SBP, arguing against a role for PPA in driving differences between individuals with high versus low SBP.

Our data should be interpreted in light of their limitations. First, because of the cross‐sectional nature of this study, it was not possible for us to infer causality. Second, use of ANCOVA models in Tables 2 and 3 considered only limited confounding factors and may be less reliable than multivariable regression models in identifying hemodynamic mechanisms relating to elevated SBP. Nevertheless, both approaches supported the observation that PVR was a key hemodynamic mechanism underlying differences in SBP in the current study. We acknowledge, however, that we did not assess the influence of biochemical variables on BP in the current analyses. The inert gas rebreathing method used in the present study yielded somewhat high values of stroke volume and cardiac output. Nevertheless, the method has been externally validated,^8^, ^9^, ^10^ and higher values are observed with subjects resting supine (as in the current study), rather than seated or standing, because of differences in venous return (preload).^11^ Moreover, we have previously compared this method with another noninvasive method based on bioreactance^11^ and observed values of cardiac output and stroke volume that were similar in magnitude to the rebreathing method described herein. We did not assess the influence of biochemical variables in our study. Finally, although regular physical activity may be associated with favorable alterations in resistance vessel structure and function, we did not have detailed enough data with which to examine physical activity as a potential modulator of vascular adaptation in this study.

To conclude, in the present study, we have demonstrated that PVR is the major hemodynamic mechanism underlying differences in SBP for a given level of PP in both male and female young adults. The presence of a high SBP may, therefore, suggest an impaired ability for the resistance vasculature to adapt appropriately, and this inability to adapt is associated with altered vascular structure.

## Appendix

### The Enigma Study Investigators

Samantha Benedict, John Cockcroft, Zahid Dhakam, Lisa Day, Stacey Hickson, Kaisa Maki‐Petaja, Barry McDonnell, Carmel McEniery, Jessica Middlemiss, Karen Miles, Maggie Munnery, Pawan Pusalkar, Christopher Retallick, Ramsey Sabit, James Sharman, Jane Smith, Jean Woodcock‐Smith, Edna Thomas, Sharon Wallace, Ian Wilkinson, and Yasmin.

## Sources of Funding

Data collection for this work was funded by the British Heart Foundation (PG03/050/15366 and FS/06/005). This work was funded, in part, by the National Institute for Health Research (NIHR) Cambridge Biomedical Research Centre. The views expressed are those of the authors and not necessarily those of the NIHR. The authors appreciate the financial support provided by China Scholarship Council (Grant No. 201806260070) for Dr Yu and acknowledge help from Fondazione per la Ricerca Biomedica Cardiovascolare Foundation.

## Disclosures

None.

## Supporting information


**Tables S1–S6**
