# Supplementary material for: Role of Vascular Adaptation in Determining Systolic Blood Pressure in Young Adults
Source: J Am Heart Assoc. 2020 Mar 30;9(7):e014375. doi: 10.1161/JAHA.119.014375 (PMC7428627; doi:10.1161/JAHA.119.014375)
Supplement: Supplementary file 1 — Tables S1–S6 [file JAH3-9-e014375-s001.pdf]

SUPPLEMENTAL MATERIAL

Table S1. Minimum and maximum values of PP for each quartile and of SBP for each tertile in males.

Range of PP for each Quartile, mmHg											
**Q1 (n=383)**			**Q2 (n=391)**			**Q3 (n=370)**			**Q4 (n=395)**		
27-48			49-54			55-60			61-97		
**Range of SBP for each Tertile, mmHg**											
**T1 (n=130)**	**T2 (n=126)**	**T3 (n=127)**	**T1 (n=131)**	**T2 (n=127)**	**T3 (n=133)**	**T1 (n=122)**	**T2 (n=123)**	**T3 (n=125)**	**T1 (n=126)**	**T2 (n=139)**	**T3 (n=130)**
85-111	112-119	120-156	103-117	118-124	125-157	102-123	124-129	130-157	113-130	131-138	139-188

Table S2. Minimum and maximum values of PP for each quartile and of SBP for each tertile in females.

Range of PP for each Quartile, mmHg											
Q1 (n=427)			Q2 (n=335)			Q3 (n=396)			Q4 (n=406)		
12-37			38-41			42-46			47-109		
**Range of SBP for each Tertile, mmHg**											
**T1 (n=137)**	**T2 (n=145)**	**T3 (n=14)**	**T1 (n=121)**	**T2 (n=96)**	**T3 (n=118)**	**T1 (n=141)**	**T2 (n=124)**	**T3 (n=131)**	**T1 (n=134)**	**T2 (n=138)**	**T3 (n=134)**
77-98	99-105	106-145	89-104	105-109	110-149	92-108	109-114	115-163	100-114	115-123	124-187

Table S3. Levels of variables in each group by quartiles of PP and tertiles of SBP in males.

	Quartile 1			Quartile 2			Quartile 3			Quartile 4			ANCOVA P
	Tertile 1	Tertile 2	Tertile 3	Tertile 1	Tertile 2	Tertile 3	Tertile 1	Tertile 2	Tertile 3	Tertile 1	Tertile 2	Tertile 3	
**Demographic and biochemical variables**													
Age, year	22±5	25±7†	27±7†‡	22±5	23±6	26±7†‡	21±4	22±5	24±6†‡	21±3	22±5†	24±6†‡	<0.001
Height, m	1.76±0.08	1.77±0.07	1.77±0.07	1.79±0.06	1.78±0.07	1.79±0.07	1.80±0.06	1.80±0.07	1.78±0.07	1.81±0.06	1.80±0.07	1.80±0.07	<0.001
Weight, kg	69.0±12.8	75.2±13.8†	82.0±16.7†‡	71.3±11.1	75.1±13.5	83.4±15.7†‡	73.8±10.5	77.2±10	83.5±14.8†‡	77.2±9.8	80.8±12.5	85.9±15.6†‡	<0.001
Body mass index, kg/m^2	22.4±3.8	24.0±4.2†	26.1±4.8†‡	22.2±3.1	23.6±3.8†	26.1±4.4†‡	22.8±2.8	23.9±3.05†	26.3±4.31†‡	23.6±2.5	24.9±3.7†	26.5±4.5†‡	<0.001
Body surface area, m^2	1.84±0.18	1.92±0.17†	1.99±0.20†‡	1.89±0.15	1.92±0.18	2.01±0.19†‡	1.92±0.14	1.96±0.13	2.01±.18†‡	1.97±0.14	2.00±0.16	2.05±0.18†‡	<0.001
Total cholesterol, mmol/L	3.99±0.84	4.17±0.93	4.59±1.13†‡	3.73±0.80	4.02±0.79†	4.46±1.06†‡	3.74±0.65	4.04±0.83†	4.40±0.99†‡	3.83±0.89	3.95±0.86	4.33±1.13†‡	<0.001
LDL cholesterol, mmol/L	2.29±0.77	2.42±0.78	2.71±1.00†‡	2.02±0.64	2.24±0.66	2.52±0.93†‡	2.09±0.56	2.21±0.72	2.49±0.85†‡	2.10±0.76	2.15±0.75	2.42±1.05†‡	0.003
HDL cholesterol, mmol/L	1.33±0.31	1.34±0.28	1.29±0.35	1.35±0.32	1.34±0.29	1.38±0.48	1.31±0.27	1.38±0.32	1.25±0.29‡	1.30±0.30	1.30±0.35	1.36±0.40	0.12
Triglycerides, mmol/L	0.93±0.51	1.10±0.78	1.37±0.92†‡	0.94±0.69	1.06±0.72	1.36±1.21†‡	0.95±0.66	1.09±0.90	1.56±1.36†‡	1.18±0.85	1.11±0.64	1.39±1.01‡	<0.001
Glucose, mmol/L	4.52±0.80	4.59±0.69	4.81±0.87†	4.64±0.69	4.74±0.70	4.73±0.77	4.85±0.76	4.76±0.81	4.77±0.65	4.82±0.84	4.89±0.79	4.98±0.76	<0.001
**Haemodynamic variables**													
Brachial SBP, mmHg	106±5	115±2†	127±8†‡	113±3	121±2†	131±7†‡	119±3	126±2†	137±7†‡	125±4	134±2†	149±10†‡	<0.001
Brachial DBP, mmHg	63±5	71±4†	82±8†‡	62±3	69±2†	79±7†‡	62±4	69±2†	80±7†‡	62±4	68±5†	79±9†‡	<0.001
Brachial PP, mmHg	43±4	44±4†	45±3†‡	51±2	52±2	52±2	57±2	58±2	58±2	64±3	67±4†	70±8†‡	<0.001
Central SBP, mmHg	91±5	99±4†	112±9†‡	95±4	102±3†	113±9†‡	98±4	105±3†	117±8†‡	101±4	109±4†	123±11†‡	<0.001
Central PP, mmHg	27±3	28±3†	29±3†‡	32±3	32±3	33±4	35±3	35±3	36±3	39±3	40±4	42±6†‡	<0.001
MAP, mmHg	76±5	84±4†	96±9†‡	77±5	84±3†	95±8†‡	78±5	85±3†	97±8†‡	79±5	86±5†	99±10†‡	<0.001
Heart rate, beats/min	61±10	65±10†	70±10†‡	63±11	66±9	67±13†	63±10	65±9	69±11†‡	63±10	66±12	70±13†‡	<0.001
PPA	1.62±0.14	1.61±0.13	1.58±0.18	1.63±0.12	1.63±0.13	1.60±0.17	1.65±0.12	1.65±0.11	1.63±0.13	1.66±0.11	1.68±0.11	1.66±0.15	<0.001
Adjusted PPA*	1.63±0.10	1.62±0.12	1.59±0.11	1.63±0.12	1.62±0.13	1.62±0.12	1.64±0.12	1.65±0.12	1.62±0.12	1.65±0.11	1.67±0.12	1.64±0.11‡	<0.001
AIx, %	1.12±10.61	3.78±10.43	6.84±12.95†	-1.39±9.44	-0.21±10.68	4.74±12.74†‡	-2.01±11.32	-1.81±10.81	2.84±10.50†‡	-3.91±10.57	-4.61±10.88	-0.01±12.56†‡	<0.001
Adjusted AIx*, %	0.30±11.29	2.19±10.21	5.41±10.42†	-1.03±10.30	-0.13±10.26	3.17±10.26†‡	-0.93±10.27	-0.93±10.20	2.96±10.17†‡	-2.03±11.22	-3.54±10.26	1.19±10.95†‡	<0.001
aPWV, m/s	5.47±0.77	5.93±0.86†	6.44±1.18†‡	5.50±0.76	5.89±0.81†	6.27±0.89†‡	5.44±0.76	5.84±0.76†	6.36±0.97†‡	5.49±0.71	5.82±0.95†	6.63±1.23†‡	<0.001
Adjusted aPWV§, m/s	6.17±1.14	5.95±0.84	5.81±1.01	5.98±0.92	5.96±0.82	5.79±0.89	5.91±0.89	5.97±0.82	5.85±0.92	6.10±1.01	5.88±0.83	5.92±1.04	0.76
Cardiac output, L/min	6.98±1.65	7.46±1.90	7.68±2.01†	7.68±1.72	8.04±2.11	7.99±2.15	8.41±2.06	8.50±2.13	8.55±1.94	9.21±2.02	8.89±1.84	9.56±2.05‡	<0.001
Cardiac index, $L/min/m^2$	3.80±0.83	3.90±0.91	3.87±0.98	4.07±0.90	4.18±1.01	3.97±1.03	4.37±0.97	4.33±1.03	4.26±0.97	4.67±0.96	4.45±0.90	4.67±0.98	<0.001
Stroke volume, mL/beat	103±25	104±31	99±28	106±24	108±30	106±29	117±33	112±31	109±26	124±32	120±30	121±30	<0.001
Stroke volume index, $mL/beat/m^2$	56±13	54±15	50±13†‡	56±12	56±14	53±13	61±16	57±15	54±12†	63±15	60±14	59±15	<0.001
PVR, $dyn{\cdot}s{\cdot}cm^{-5}$	921±237	963±266	1075±305†‡	842±208	907±318	1052±45†‡	787±214	851±234	957±248†‡	722±202	810±185†	874±231†‡	<0.001
TAC, mL/beat/mmHg	3.93±1.00	3.85±1.20	3.48±1.04†‡	3.41±0.74	3.38±0.93	3.23±0.92	3.37±0.92	3.23±0.88	3.09±0.70†	3.23±0.82	3.01±0.71	2.85±0.71†	<0.001
Adjusted TAC₸, mL/beat/mmHg	3.92±0.92	3.89±0.90	3.58±0.91	3.40±0.90	3.42±0.89	3.23±0.89	3.35±0.91	3.22±0.88	3.10±0.89	3.14±0.90	2.96±0.90	2.84±0.92†	<0.001

Values are means ± SD. ANCOVA was performed among all groups and multiple comparisons with Bonferroni method were performed between SBP groups within each quartile of PP. † P<0.05 versus tertile 1; ‡ P<0.05 versus tertile 2. * Further adjusted for height and heart rate. § Further adjusted for mean arterial pressure and heart rate; ₸ Further adjusted for body mass index and heart rate. LDL, low density lipoprotein; HDL, high density lipoprotein; SBP, systolic blood pressure; DBP, diastolic blood pressure; PP, pulse pressure; MAP, mean arterial pressure; PPA, pulse pressure amplification; AIx, augmentation index; aPWV, aortic pulse wave velocity; PVR, peripheral vascular resistance; TAC, total arterial compliance.

Table S4. Levels of variables in each group by quartiles of PP and tertiles of SBP in females.

	Quartile 1			Quartile 2			Quartile 3			Quartile 4			ANCOVA P
	Tertile 1	Tertile 2	Tertile 3	Tertile 1	Tertile 2	Tertile 3	Tertile 1	Tertile 2	Tertile 3	Tertile 1	Tertile 2	Tertile 3	
**Demographic and biochemical variables**													
Age, year	22±4	23±5†	24±6†‡	23±6	23±6	24±6†‡	22±5	22±6	26±7†‡	22±5	22±5†	26±7†‡	<0.001
Height, m	1.63±0.07	1.64±0.06	1.63±0.07	1.65±0.07	1.66±0.07	1.64±0.07	1.66±0.07	1.66±0.06	1.66±0.07	1.67±0.06	1.68±0.07	1.65±0.08‡	<0.001
Weight, kg	57.4±11.2	60.7±11.2	63.7±13.3†	60.5±8.8	63.2±9.9	64.1±10.9†	60.5±8.7	61.8±8.6	68.8±15.4†‡	63.8±7.9	66.5±10.4	71.5±17.0†‡	<0.001
Body mass index, kg/m^2	21.5±3.8	22.6±3.7	23.9±4.5†‡	22.2±2.8	22.8±3.34	23.9±3.9†	21.9±2.7	22.6±3.2	25.0±5.1†‡	22.8±2.7	23.6±3.6	26.3±5.9†‡	<0.001
Body surface area, m^2	1.61±0.16	1.65±0.15	1.68±0.17†	1.66±0.13	1.70±0.14	1.69±0.15	1.67±0.14	1.68±0.12	1.76±0.19†‡	1.72±0.11	1.75±0.5	1.78±0.20†	<0.001
Total cholesterol, mmol/L	4.13±0.84	4.36±1.01	4.35±0.79	4.19±0.81	4.12±0.67	4.24±0.87	4.05±0.87	4.24±0.87	4.53±1.02†‡	4.21±0.80	4.22±0.83	4.43±0.86	0.04
LDL cholesterol, mmol/L	2.30±0.64	2.46±0.96	2.46±0.71	2.34±0.67	2.23±0.60	2.30±0.73	2.18±0.70	2.31±0.79	2.61±0.95†‡	2.28±0.69	2.29±0.73	2.49±0.79	0.03
HDL cholesterol, mmol/L	1.54±0.40	1.56±0.44	1.50±0.40	1.54±0.34	1.55±0.40	1.56±0.42	1.56±0.36	1.55±0.37	1.53±0.44	1.54±0.36	1.57±0.38	1.50±0.42	0.77
Triglycerides, mmol/L	0.77±0.40	0.89±0.50	0.94±0.52†	0.81±0.47	0.86±0.42	0.91±0.49	0.76±0.39	0.92±0.53†	0.99±0.62†	0.93±0.60	0.93±0.54	1.10±0.72	<0.001
Glucose, mmol/L	4.36±0.75	4.50±0.65	4.56±0.78	4.40±0.69	4.64±0.72†	4.56±0.67	4.51±0.76	4.55±0.73	4.77±0.81†	4.50±0.71	4.55±0.79	4.72±0.83	<0.001
**Haemodynamic variables**													
Brachial SBP, mmHg	93±5	102±2†	113±7†‡	100±3	107±2†	117±7†‡	104±3	111±2†	123±8†‡	110±4	119±2†	138±12†‡	<0.001
Brachial DBP, mmHg	62±5	68±3†	78±8†‡	61±4	67±2†	78±7†‡	60±4	67±2†	78±9†‡	60±4	68±4†	83±11†‡	<0.001
Brachial PP, mmHg	32±3	34±3†	34±3†	40±1	40±1	40±1	44±1	44±1	44±1	50±3	51±4	55±8†‡	<0.001
Central SBP, mmHg	82±5	90±3†	102±8†‡	86±5	92±3†	104±9†‡	89±4	95±3†	108±11†‡	92±4	100±5†	121±15†‡	<0.001
Central PP, mmHg	20±3	22±3†	22±3†	25±3	25±3	26±4†‡	27±3	27±3	29±4†‡	31±3	31±4	37±8†‡	<0.001
MAP, mmHg	72±5	79±5†	90±80†‡	73±4	79±2†	91±8†‡	74±5	81±3†	92±10†‡	75±4	83±4†	102±13†‡	<0.001
Heart rate, beats/min	64±11	67±10	70±11†‡	65±9	68±11	70±11†	65±11	67±11	71±12†	64±10	68±11†	73±11†‡	<0.001
PPA	1.57±0.17	1.59±0.17	1.55±0.19	1.62±0.17	1.63±0.15	1.55±0.19†‡	1.62±0.16	1.64±0.15	1.56±0.21†‡	1.63±0.13	1.64±0.17	1.50±0.23†‡	<0.001
Adjusted PPA*	1.58±0.16	1.60±0.14	1.54±0.15‡	1.64±0.14	1.61±0.15	1.55±0.14†‡	1.61±0.15	1.62±0.14	1.58±0.15	1.62±0.15	1.61±0.15	1.51±0.15†‡	<0.001
AIx, %	6.76±12.94	7.30±12.29	12.16±12.29†‡	4.91±12.05	4.50±12.42	11.85±12.93†‡	3.25±11.59	4.36±11.96	10.72±13.10†‡	1.17±11.95	1.63±13.18	15.18±15.16†‡	<0.001
Adjusted AIx*, %	6.00±11.70	6.38±10.72	12.39±11.32†‡	3.86±10.87	5.42±10.79	11.38±10.85†‡	3.99±10.73	5.35±10.81	9.46±10.92†‡	2.55±11.69	4.16±10.96	14.39±12.04†‡	<0.001
aPWV, m/s	5.27±0.94	5.49±0.90	5.80±0.82†‡	5.30±0.66	5.31±0.61	5.91±0.99†‡	5.20±0.73	5.46±0.84†	6.09±1.11†‡	5.25±0.70	5.56±0.72†	6.48±1.23†‡	<0.001
Adjusted aPWV§, m/s	5.84±1.05	5.61±0.78	5.58±0.84	5.67±0.85	5.43±0.78	5.54±0.83	5.60±0.83	5.56±0.77	5.58±0.86	5.70±0.93	5.61±0.78	5.63±1.15	0.31
Cardiac output, L/min	5.63±1.29	5.76±1.26	6.06±1.36†	6.13±1.32	6.43±1.41	6.41±1.41	6.26±1.50	6.69±1.54	6.64±1.75	6.66±1.56	7.10±1.32	6.96±1.71	<0.001
Cardiac index, L/min/m^2	3.48±0.69	3.48±0.69	3.61±0.77	3.70±0.80	3.77±0.77	3.80±0.83	3.74±0.80	3.98±0.87	3.79±0.95	3.88±0.88	4.06±0.73	3.93±0.94	<0.001
Stroke volume, mL/beat	80±19	78±16	77±17	84±19	86±20	82±20	90±24	87±21	85±25	92±24	91±19	86±25	<0.001
Stroke volume index, mL/beat/m^2	50±11	47±9	46±9†	51±11	50±11	48±12	53±13	52±12†	49±13	54±14	52±11	49±14†	<0.001
PVR, dyn·s·cm^{-5}	1070±255	1157±286†	1244±298†‡	998±248	1045±281	1194±309†‡	994±228	1016±237	1187±361†‡	952±248	972±218	1252±399†‡	<0.001
TAC, mL/beat/mmHg	3.97±0.95	3.62±0.77†	3.49±0.99†	3.45±0.78	3.50±0.78	3.13±0.84†‡	3.30±0.91	3.18±0.83†	2.93±0.89	3.01±0.79	2.90±0.67	2.39±0.49†‡	<0.001
Adjusted TAC₸, mL/beat/mmHg	3.98±0.94	3.64±0.84†	3.50±0.96†	3.46±0.85	3.50±0.86	3.16±0.86‡	3.29±0.88	3.17±0.86	2.97±0.88	2.97±0.93	2.85±0.86	2.41±0.98†‡	<0.001

Values are means ± SD. ANCOVA was performed among all groups and multiple comparisons with Bonferroni method were performed between SBP groups within each quartile of PP. † P<0.05 versus tertile 1; ‡ P<0.05 versus tertile 2. * Further adjusted for height and heart rate. § Further adjusted for mean arterial pressure and heart rate; ₸ Further adjusted for body mass index and heart rate. LDL, low density lipoprotein; HDL, high density lipoprotein; SBP, systolic blood pressure; DBP, diastolic blood pressure; PP, pulse pressure; MAP, mean arterial pressure; PPA, pulse pressure amplification; AIx, augmentation index; aPWV, aortic pulse wave velocity; PVR, peripheral vascular resistance; TAC, total arterial compliance.

Table S5. The association of SBP with haemodynamic parameters in the middle two quartiles of PP in males.

Models	PP Quartile 2			PP Quartile 3		
	β	P	Model R^2	β	P	Model R^2
**Univariable linear regression analysis**						
Cardiac output	0.02	0.72	<0.001	0.02	0.67	<0.001
Stroke volume	-0.05	0.29	<0.001	-0.09	0.10	0.005
PVR	0.32	**<0.001**	0.10	0.36	**<0.001**	0.13
TAC	-0.14	**0.007**	0.02	-0.15	**0.004**	0.02
PPA	-0.15	**0.005**	0.02	-0.13	**0.02**	0.01
AIx	0.31	**<0.001**	0.09	0.26	**<0.001**	0.06
aPWV	0.43	**<0.001**	0.18	0.46	**<0.001**	0.21
**Multivariable linear regression analysis**						
Cardiac output[a]	0.05	0.30	0.24	0.03	0.57	0.22
Stroke volume[b]	-0.16	**0.001**	0.26	-0.12	**0.01**	0.23
PVR[b]	0.28	**<0.001**	0.35	0.28	**<0.001**	0.34
TAC[b]	-0.14	**0.002**	0.30	-0.08	0.11	0.28
PPA[c]	-0.12	**0.03**	0.19	-0.14	**0.01**	0.20
AIx[c]	0.26	**<0.001**	0.22	0.22	**<0.001**	0.22
aPWV[d]	0.009	0.64	0.90	0.02	0.17	0.93

Univariable and multivariable linear regression analyses were performed. [a] adjusted for age and body mass index; [b] adjusted for age, body mass index and heart rate; [c] adjusted for age, height and heart rate; [d] adjusted for age, body mass index, mean blood pressure and heart rate. β, standardized regression coefficient; PVR, peripheral vascular resistance; TAC, total arterial compliance; PPA, pulse pressure amplification; AIx, augmentation index; aPWV, aortic pulse wave velocity.

Table S6. The association of SBP with haemodynamic parameters in the middle two quartiles of PP in females.

Models	PP Quartile 2			PP Quartile 3		
	**β**	**P**	**Model R^2**	**β**	**P**	**Model R^2**
**Univariable linear regression analysis**						
Cardiac output	0.03	0.53	<0.001	0.18	**<0.001**	0.03
Stroke volume	-0.12	**0.03**	0.01	0.01	0.91	<0.001
PVR	0.41	**<0.001**	0.17	0.28	**<0.001**	0.08
TAC	-0.27	**<0.001**	0.07	-0.11	**0.048**	0.01
PPA	-0.30	**<0.001**	0.09	-0.25	**<0.001**	0.06
AIx	0.34	**<0.001**	0.11	0.32	**<0.001**	0.10
aPWV	0.44	**<0.001**	0.19	0.53	**<0.001**	0.28
**Multivariable linear regression analysis**						
Cardiac output[a]	0.03	0.59	0.05	0.13	**0.007**	0.21
Stroke volume[b]	-0.15	**0.004**	0.07	-0.06	0.18	0.19
PVR[b]	0.41	**<0.001**	0.29	0.25	**<0.001**	0.31
TAC[b]	-0.22	**<0.001**	0.17	-0.05	0.33	0.23
PPA[c]	-0.46	**<0.001**	0.24	-0.25	**<0.001**	0.19
AIx[c]	0.44	**<0.001**	0.22	0.27	**<0.001**	0.22
aPWV[d]	-0.004	0.72	0.96	0.02	0.25	0.89

Univariable and multivariable linear regression analyses were performed. [a] adjusted for age and body mass index; [b] adjusted for age, body mass index and heart rate; [c] adjusted for age, height and heart rate; [d] adjusted for age, body mass index, mean blood pressure and heart rate. β, standardized regression coefficient; PVR, peripheral vascular resistance; TAC, total arterial compliance; PPA, pulse pressure amplification; AIx, augmentation index; aPWV, aortic pulse wave velocity.
